# What Constitutes Emergent Quantum Reality? A Complex System Exploration from Entropic Gravity and the Universal Constants

**DOI:** 10.3390/e20050335

**Published:** 2018-05-02

**Authors:** Arno Keppens

**Affiliations:** Space Pole, Circular Avenue, 1180 Brussels, Belgium; arnok@oma.be; Tel.: +32-2373-0412

**Keywords:** quantum ontology, sub-quantum dynamics, micro-constituents, emergent space-time, emergent quantum gravity, entropic gravity, black hole thermodynamics

## Abstract

In this work, it is acknowledged that important attempts to devise an emergent quantum (gravity) theory require space-time to be discretized at the Planck scale. It is therefore conjectured that reality is identical to a sub-quantum dynamics of ontological micro-constituents that are connected by a single interaction law. To arrive at a complex system-based toy-model identification of these micro-constituents, two strategies are combined. First, by seeing gravity as an entropic phenomenon and generalizing the dimensional reduction of the associated holographic principle, the universal constants of free space are related to assumed attributes of the micro-constituents. Second, as the effective field dynamics of the micro-constituents must eventually obey Einstein’s field equations, a sub-quantum interaction law is derived from a solution of these equations. A Planck-scale origin for thermodynamic black hole characteristics and novel views on entropic gravity theory result from this approach, which eventually provides a different view on quantum gravity and its unification with the fundamental forces.

## 1. Introduction

Important attempts to devise an emergent quantum (gravity) theory require space-time to be discretized at the Planck scale [1]. The identification of the discrete micro-constituents of space-time is therefore one of the biggest research questions in present-day physics. Yet, if space-time is indeed an effective field, emerging from the interaction of its micro-constituents only, then quantizing some aspect of general relativity will not help us identify its fundamental degrees of freedom—by analogy, we would arrive at a theory of phonons rather than a description of the underlying atoms of the condensate [2,3,4]. For that reason, in correspondence with Oriti [5], in this work “we consider the emergence of continuum space and time from the collective behavior of discrete, pre-geometric atoms of quantum space, and (analogously consider) space-time as a kind of condensate”.

Yet, by viewing the conjectured pre-geometric atoms of quantum space as the ontological micro-constituents of our emergent reality, its effective macro-dynamics, including space and time, is expected to benefit from a complex (nonlinear) sub-quantum dynamical systems approach for its appropriate understanding in terms of the fundamental degrees of freedom. According to Ladyman et al. [6] “a complex system is an ensemble of many elements which are interacting in a disordered way, resulting in robust organization and memory”. The necessary qualitative conditions, although being not necessarily jointly sufficient, for the emergence of a complex dynamic that shows spontaneous yet persistent ordering can be correspondingly defined as “numerosity” (an ensemble of many fungible elements) and “interaction” (through direct nonlinear causality) [6].

This work hence attempts to provide a parsimonious complex systems approach, as a kind of toy model, for identifying space-time’s ontological micro-constituents and their interaction, i.e., their sub-quantum dynamics. Motivated by Occam’s razor, it is here assumed that only one type of such micro-constituents exists, and that a single interaction law connects them relationally [7]. This assumption entails that effective space-time, matter, gravity, and the other fundamental forces should emerge from the interaction, through their fundamental degrees of freedom as dynamical attributes, of the single-type micro-constituents. A number of analogue gravity models or condensed matter approaches to quantum gravity already adopt this strategy, but typically lack background-independence in their interactions [4,8]. This background-independence however is required for interactions that induce (and thus precede) the emergence of any space-time that could serve as a reference metric.

In order to arrive at a background-independent micro-constituent interaction (law) that reproduces general relativity’s dynamical space-time (including gravity) in its effective field behavior, we adopt and combine two strategies. First, motivated by the works of Jacobson [2], Padmanabhan [9], and Verlinde [10] (or see Padmanabhan [11] for more recent progress), we will conceive of gravity as a thermodynamic phenomenon or an emergent entropic force. These authors have demonstrated how Einstein’s field equations can be considered to originate from space-time’s thermodynamic degrees of freedom at a causal (black hole or holographic) horizon. In this work however, in order to identify the micro-constituents of space-time and their relation with common physical quantities, the dimensional reduction of the holographic principle as presented by ‘t Hooft [12] is generalized to non-holographic reference surfaces. It is shown that the universal constants of free space can then be related to attributes of the atoms of quantum space.

Second, a reverse-engineering argument, somewhat characteristic for complex dynamical systems approaches and encouraged by Hu [13] for emergent quantum gravity research, is used to put forward an approximation of the background-independent interaction law that connects the conjectured single-type micro-constituents of space-time: as the emergent effective field dynamics of the micro-constituents must eventually obey Einstein’s relativistic field equations [14], a micro-constituent interaction law that yields the required diffeomorphism invariant field behavior can be obtained from a solution of these equations. The resulting interaction law is however formulated within the emergent relativistic space-time framework itself, and not in a fundamental pre-space-time framework. The latter option is very much complicated by the involvement of some sort of “external time” that is tied to the pre-space-time dynamics of the micro-theory [4]. This flaw seems familiar—and acceptable—when looking at the analogous issue in perturbative string theory, see for instance Huggett and Vistarini [15].

Together, these two strategies thus allow identifying—in a first rudimentary way—the micro-constituents of space-time and their basic interaction. The explicit constituent-based complex systems approach presented in this work additionally allows deriving black hole thermodynamics in a way that is believed to be more direct and intuitive than previous accounts [16,17,18] and related aspects of entropic gravity, the latter even for non-holographic reference surfaces. Both phenomena are reproduced in terms of space-time’s micro-constituents and the number of fundamental (thermodynamic) degrees of freedom at their availability on the surface of reference. This complex toy model of quantum reality is therefore anticipated to point the way towards a more mature emergent theory of quantum gravity, while a generalization of the constituent-based origin of the gravitational field finally hints at a unification of the fundamental forces.

## 2. Constituent Identification

We initiate our complex systems-based toy model of emergent reality with a rudimentary attempt to identify space-time’s ontological micro-constituents. It is thereby assumed that only one type of such micro-constituents exists, which entails that effective space-time, matter, gravity, and the other fundamental forces should emerge from the interaction, through their attributes, of these single-type micro-constituents only. This also entails that the universal constants of free space, like the speed of light in vacuum c, the gravitational constant G, the (reduced) Planck constant $\bar{h}$, and the Boltzmann constant $k_{B}$, are expected to be in some way all related to the attributes of the micro-constituents. A direct connection between the universal constants of free space and associated space-time constituent properties is therefore derived in the following.

As space-time (curvature) and gravitational effects are unified by Einstein’s relativistic field equations, it seems evident to first establish a relationship between the mass m or energy E enclosed within a certain space-time volume V on the one hand and an invariable property (say $G_{0}$) of each of the $n_{V}$ individual space-time constituents within that volume on the other hand:

(1) $$m\propto n_{V}G_{0}.$$

Let us denote this mass and energy defining attribute, $G_{0}$, which should obviously be related to the gravitational constant, as a micro-constituent’s “gravitational presence” (this choice is elucidated later on). Yet, masses also experience their mutual full extent from a distance, i.e., without shared knowledge of their respective $n_{V}$. We must, therefore, relate the “information” about the amount of micro-constituents within the volume V to some “information” on its surface A=∂V, which is the kind of dimensional reduction that was proposed by ‘t Hooft [12] in his holographic principle. This principle is generalized to non-holographic surfaces here with the following premise: the amount of micro-constituents $n_{V}$ contained within an enclosed space-time volume V is proportional to the amount of micro-constituents $n_{A}$ that overlaps with the surface A=∂V of that volume: $n_{V}\propto n_{A}$*.* As a result, one can rewrite Equation (1) as:

(2) $$m\propto n_{A}G_{0}.$$

Relating the above to common physical quantities can be achieved by use of straightforward dimensional analysis. By simply rearranging the unit dimensions of G one has:

(3) $$m\propto \frac{c^{3}}{G}\Delta t.$$

By combination of Equations (2) and (3), and thereby taking $\Delta t=t_{P}$ to explicitly relate the constituents to the Planck scale (and unit system), one can identify each mass as follows:

(4) $$m\equiv n_{A}G_{0}t_{P}$$

with $G_{0}\propto c^{3}/G$ from Equation (3). Equation (4) implies:

(5) $$\begin{array}{ccc} \begin{array}{c} m_{0}\\ E_{0} \end{array} & \begin{array}{c} =\\ = \end{array} & \begin{array}{c} G_{0}l_{P}/c\\ G_{0}l_{P}c \end{array} \end{array}$$

so that we can write $m=n_{A}m_{0}$ and $E=n_{A}E_{0}$ with $m_{0}$ and $E_{0}$ the rather abstract unit mass and unit energy that are associated with the exchange of a single space-time micro-constituent through the surface A, respectively. In the following, $n_{A}$ is replaced by n, as always the micro-constituents on the reference surface are intended.

Up to this point, our analysis has been limited to linear relationships in terms of the numbers of micro-constituents. This changes when considering temperature T and entropy S that both depend on a system’s thermodynamic degrees of freedom. Motivated by the entropic gravity argumentation from Padmanabhan [9] and Verlinde [10] for holographic surfaces, yet keeping our non-holographic premise and Equation (2) in mind, we here apply the equipartition theorem to the generalized reference surface A (assuming that it also holds approximately for non-trivial energy distributions in quantum systems). The equipartition theorem then states that the energy $nE_{0}$ of V, because of its representation by the n micro-constituents at the surface A of V, is equally distributed over all degrees of freedom N on A, or $E=nE_{0}=Nk_{B}T/2$, which immediately results in:

(6) $$T=\frac{2nE_{0}}{Nk_{B}}.$$

The connection between temperature and entropy as conjugate thermodynamic variables through T=ΔE/ΔS, which is discretized because of the finite-sized micro-constituents, moreover yields:

(7) $$\Delta S=\frac{k_{B}N}{2}\frac{\Delta n}{n}.$$

By direct integration for constant N, i.e., over the reference surface A, Equation (7) becomes:

(8) $$S=\frac{k_{B}N}{2}\ln\left(n\right)$$

so that, on the Planck unit scale, $S_{P}=k_{B}\ln\left(2\right)$ bit or $S_{P}=k_{B}$ nit (as required by definition) only when n=N=2. This entails that a surface enclosing a single Planck mass exchanges two space-time micro-constituents with the outer environment during a single Planck time interval or $\sim 10^{43}$ constituents over a second. The entropy associated with a single constituent occupying one fundamental degree of freedom S (n=1,N=1) obviously equals zero, yet one can define $S_{0}=S\;\left(n=2,N=1\right)=k_{B}/2$ nit as a unit simplification, wherefrom, upon insertion into Equations (6) and (8) respectively:

(9) $$T=\frac{n}{N}\frac{E_{0}}{S_{0}}=\frac{n}{N}T_{0}$$

and

(10) $$S=S_{0}N\ln\left(n\right).$$

Comparison with the Boltzmann formula $S=k_{B}\ln\left(\Omega \right)$ shows that the number of microstates Ω that corresponds with a given macrostate encompassing N surface degrees of freedom for n micro-constituents is given by $\Omega =n^{N}$ as one would expect.

By combining $m_{P}=2G_{0}l_{P}/c$ with the Planck definitions of mass $m_{P}=\sqrt{\bar{h}c/G}$ and length $l_{P}=\sqrt{\bar{h}G/c^{3}}$ [19], one obtains:

(11) $$\begin{array}{ccc} G & = & c^{3}/2G_{0}\\ \bar{h} & = & 2G_{0}l_{P}^{2} \end{array}.$$

As summarized in Table 1, the above allows translating the universal constants of free space into four attributes of space-time’s micro-constituents and corresponding constituent units. Note that products of constituent units of complementary variables, like time and energy or position and momentum, immediately yield $G_{0}l_{P}^{2}=\bar{h}/2$. This result suggests a direct connection between the discreteness of the micro-constituents, forcing measurement outcomes to refer to an integer amount of constituents, and the Heisenberg uncertainty relations [20].

## 3. Constituent Interaction

Inventing a valid constant translation and unit redefinition can be done in numerous ways and is therefore not highly remarkable. The translation developed above however aims at getting as close as possible to the very nature of reality by considering the attributes that are allocated to individual micro-constituents of space-time as its basis. The next step in our search for a complex theory of quantum gravity would then be to connect the constituent properties defined in Table 1 by an interaction law that yields an effective dynamics in agreement with present-day physics theories. From a gravitational perspective, the emergent effective field dynamics must obey Einstein’s field equations of general relativity [14]. Motivated by Hu [13], a relational micro-constituent interaction law that yields diffeomorphism invariant fielding behavior, yet formulated within the emergent relativistic space-time framework, can therefore be derived from a solution of these equations.

In the weak field approximation (neglecting the exact Schwarzschild solution to simplify the discussion), where the metric tensor is defined as a small perturbation (≪1) on the Minkowski metric due to a mass M, the line element ds at a distance R from M is given by [14]:

(12) $$ds^{2}\approx \left(1-\frac{2GM}{c^{2}R}\right)c^{2}dt^{2}-\left(1+\frac{2GM}{c^{2}R}\right)dl^{2}$$

with $dl^{2}=dx^{2}+dy^{2}+dz^{2}$. As the micro-constituents move at the speed of light (see Table 1), the effective space-time constituent speed, denoted as $c^{\prime }$, is then given by ds=0 or:

(13) $$c^{\prime }\equiv \frac{dl}{dt}\approx c\left(1-\frac{2GM}{c^{2}R}\right).$$

In constituent units, this becomes:

(14) $$c^{\prime }\approx c\left(1-\frac{l_{P}}{R}n_{M}\right)\equiv c\left(1-\rho_{r}\right)$$

whereby $\rho_{r}\equiv n_{M}l_{P}/R=n_{M}/R_{P}$ is defined as the “radial constituent density” i.e., the amount of micro-constituents exchanged by M through the surface $4\pi R^{2}$ relative to the distance R from M in units $l_{P}$, which reflects gravity’s spherical isotropy.

Equation (14) shows that the constituent speed as measured in a non-inertial coordinate system at distance R from M indeed decreases with declining R [21,22]. Stated differently, there exists an effective index of refraction $\eta \approx {\left(1-\rho_{r}\right)}^{-1}$ with $\rho_{r}$ representing an effective local constituent density (field). According to the same non-inertial coordinate system, the space-time constituents must therefore undergo an acceleration $a_{0}$ given by $dc^{\prime }/dt\approx 2GM/R^{2}$ or $dc^{\prime }/dt\approx c^{2}l_{P}n_{M}/R^{2}$ in constituent units, wherefrom:

(15) $$a_{0}\approx \frac{4\pi c^{2}}{l_{P}}\frac{n_{M}}{N}$$

provided that $N=A/l_{P}^{2}=4\pi R^{2}/l_{P}^{2}=4\pi R_{P}^{2}$ here. This identity however has been derived by Padmanabhan for any diffeomorphism invariant theory [23,24]. By the very conception of mass in Equation (4), $n_{M}$ refers to the number of space-time constituents intersecting a spherical surface with radius R, entailing that N must indeed equal the number of fundamental degrees of freedom on this same surface in constituent units. Most importantly, Equation (15) translates the presence of a remote massive object M into a local experience (and interaction) of gravitational presences at distance R from M, i.e., into a function of the amount of micro-constituents $n_{M}$ relative to the number of degrees of freedom N at their availability (also see next section). There is no reference to any prior geometry, or in other words Equation (15) is a background-independent constituent interaction law.

Black hole thermodynamics follows straightforwardly [25]: A spherical surface with radius $R_{S}$ enclosing a compound massive object M will have $c^{\prime }\to 0$ when its radial constituent density $\rho_{r}=n_{M}l_{P}/R_{S}\to 1$ according to Equation (14). This means that the escape velocity from M equals c at $R_{S}=n_{M}l_{P}$, which exactly matches the Schwarzschild radius $R_{S}=2GM/c^{2}$ in constituent units. The corresponding number of degrees of freedom of the spherical reference surface at $R_{S}$ is hence given by $N_{S}=4\pi R_{S}^{2}/l_{P}^{2}=4\pi n_{M}^{2}$, entailing that $\Delta S_{BH}=2\pi k_{B}n_{M}\Delta n_{M}$ from Equation (7). Integration yields

(16) $$S_{BH}=\pi k_{B}n_{M}^{2}=\frac{k_{B}N_{S}}{4}$$

in agreement with Hawking’s black hole entropy expression [26]. The Bekenstein–Hawking black hole radiation temperature $T_{BH}$ can be determined most easily from Equation (9):

(17) $$T_{BH}=\frac{n_{M}}{N_{S}}T_{0}=\frac{T_{0}}{4\pi n_{M}}$$

which is identical to the result obtained by inserting the constant translations proposed in the previous section into the regular Bekenstein–Hawking expression [27,28]. This constituent-based origin for thermodynamic black hole characteristics is however considered to be more direct and intuitive than earlier accounts [16,17,18].

## 4. Entropic Gravity

Based predominantly on the works by Padmanabhan [9] and Verlinde [10], we attempt to relate the previous outcomes back to the interpretation of gravity as an entropic force, yet generalized to non-holographic reference surfaces. Adopting Verlinde’s classical approach first, consider the force F induced by a mass $M=n_{M}m_{0}$ onto a mass $m=n_{m}m_{0}$ (and vice-versa) at distance R, which is according to Newton’s law and in constituent units given by:

(18) $$F=\frac{G_{0}l_{P}^{2}c}{2R^{2}}n_{m}n_{M}.$$

This force induces an acceleration $a_{m}$ on m of the size F/m or:

(19) $$a_{m}=\frac{2\pi c^{2}}{l_{P}}\frac{n_{M}}{N}$$

which differs from Equation (15) only by a factor of two, as one would expect for a calculation that omits relativity’s temporal perturbation of the space-time metric [22]. Equation (19), however, immediately reproduces the Unruh temperature expression upon insertion of Equation (6) [29]. This straightforward connection in constituent units again supports the idea to regard gravity as a thermodynamic phenomenon or an emergent entropic force, as suggested before.

According to Verlinde, one can write the gravitational pull induced by M on m also as [10]:

(20) $$F={\left(\frac{\Delta E}{\Delta R}\right)}_{m}={\left(\frac{\Delta E}{\Delta S}\right)}_{m}{\left(\frac{\Delta S}{\Delta R}\right)}_{m}$$

with immediately from Equation (6) for the reference surface temperature induced by m:

(21) $${\left(\frac{\Delta E}{\Delta S}\right)}_{m}=\frac{2G_{0}l_{P}c}{k_{B}}\frac{n_{m}}{N}.$$

Also according to Verlinde, the last factor in Equation (20), being the entropy variation ΔS at the location of m that corresponds to a variation in the distance ΔR between the two masses, can be considered from the Bekenstein conjecture [27]: The effective distance shift that is needed to add one unit of entropy $\Delta S=k_{B}$ to the holographic reference surface at m equals the Compton wavelength $\bar{h}/mc=2l_{P}/n_{m}$ wherefrom (with subscript B to denote the Bekenstein-based approach):

(22) $${\left(\frac{\Delta S}{\Delta R}\right)}_{B}=\frac{k_{B}n_{m}}{2l_{P}}.$$

However, inserting Equations (21) and (22) into Equation (20) only yields Equations (18) and (19) apart from an unexplained factor $2\pi n_{M}/n_{m}$ or $4\pi n_{M}/n_{m}$ with respect to the general relativistic Equation (15). Such dissimilarity, which must be due to the Bekenstein conjecture, has also been observed by Verlinde in regular units [10]. Verlinde nevertheless uses his version of Equation (22) to relate the classical gravitational acceleration with a mass-induced entropy gradient. The same result (still by a factor $2\pi n_{M}/n_{m}$) is immediately obtained here by inserting the latter identity into Equation (19):

(23) $$a_{m,B}=\frac{4\pi c^{2}}{k_{B}N}\frac{n_{M}}{n_{m}}{\left(\frac{\Delta S}{\Delta R}\right)}_{B}.$$

For a general description that is not bound to a holographic scenario, Equation (8) instead of the Bekenstein conjecture should be used as a starting point for determining the distance-dependent entropy gradient that is induced by the mass M. In that case, with $n_{M}$ being independent of R:

(24) $${\left(\frac{\Delta S}{\Delta R}\right)}_{C}=\frac{k_{B}}{2}\frac{8\pi R}{l_{P}^{2}}\ln\left(n_{M}\right)=\frac{2S}{R}$$

whereby the subscript C stresses the constituent-based approach, so that:

(25) $$a_{m,C}=\pi c^{2}\frac{n_{M}}{N}\frac{R_{P}}{S}{\left(\frac{\Delta S}{\Delta R}\right)}_{C}$$

One can immediately reproduce the results by Padmanabhan [9] and Verlinde [10] by insertion of the Schwarzschild solutions $R_{S}=n_{M}l_{P}$ and $S_{BH}=\pi k_{B}n_{M}^{2}$ into Equations (24) and (25) respectively, yielding (with subscript S for Schwarzschild):

(26) $${\left(\frac{\Delta S}{\Delta R}\right)}_{S}=\frac{2S_{BH}}{R_{S}}=\frac{2\pi k_{B}n_{M}}{l_{P}}$$

which indeed differs from Equation (22) by a factor $4\pi n_{M}/n_{m}$ as anticipated, and consequently for the entropy-induced acceleration:

(27) $$a_{m,S}=\frac{c^{2}}{k_{B}N}{\left(\frac{\Delta S}{\Delta R}\right)}_{S}.$$

The entropic interpretation of gravitational pull can however be simplified by definition of an “informational constituent density” $\rho_{i}=n_{M}/N$, which is like a temperature according to Equation (9), as the amount of micro-constituents $n_{M}$ that is exchanged by M relative to the number of degrees of freedom N at their availability on a spherical reference surface at distance R. Taking into account again that $N=4\pi R_{P}^{2}$, the gradient of $\rho_{i}$ as experienced by m is given by:

(28) $$\frac{\Delta \rho_{i}}{\Delta R}=\frac{\Delta }{\Delta R}\left(\frac{n_{M}l_{P}^{2}}{4\pi R^{2}}\right)=-\frac{2\rho_{i}}{R}.$$

Note the similarity with the entropic gradient in Equation (24). As a result, the gravitational acceleration is very straightforwardly considered as being induced by an informational constituent density gradient also in Equation (19):

(26) $$a_{m}=-\pi c^{2}R_{P}\frac{\Delta \rho_{i}}{\Delta R}.$$

For the relativistic space-time constituents interacting through Equation (15), this means that:

(30) $$a_{0}\approx -2\pi c^{2}R_{P}\frac{\Delta \rho_{i}}{\Delta R}=-c^{2}\frac{\Delta \rho_{r}}{\Delta R}.$$

corresponding elegantly with a gravitational potential $\varphi =c^{2}n_{M}/R_{P}$.

The interpretation of entropic gravity by Padmanabhan [9] and Verlinde [10] in terms of a temperature-induced entropy change on a holographic screen due to a mass m (the Bekenstein conjecture), which causes an entropy gradient, which causes acceleration, is thus replaced here by an interpretation of gravitational pull in terms of micro-constituent density gradients: Each mass can be experienced by a remote mass, due to the experience of an effective (informational) constituent density gradient, which can be expressed as a temperature or entropy gradient, and which causes an acceleration. Although technical differences are small, the latter interpretation is believed to provide an improved conceptual understanding of emergent quantum gravity in terms of space-time’s micro-constituents and the fundamental degrees of freedom at their availability. Further entropic gravity generalizations by Padmanabhan [9] and Verlinde [10] still hold true, while a covariant Lagrangian version has been provided by Hossenfelder [30]. Relating the micro-constituent-based interpretation of entropic gravity as presented here to promising studies in entropic cosmology [31,32,33,34,35] is subject of ongoing research.

## 5. Discussion

From the necessary conditions for the emergence of a complex dynamical system, it has been conjectured that reality is identical to a sub-quantum dynamics of indistinguishable yet ontological micro-constituents that are connected by a single interaction law. In order to arrive at a first toy-model identification of these micro-constituents, two strategies have been combined. First, it is obvious that masses, which can only consist of constituent collections, require a means to fully experience each other from a distance, i.e., some kind of information about the presence and extent of each mass must be remotely available. This kind of dimensional reduction of information has been achieved from a micro-constituent-based generalization of the holographic principle within a thermodynamic interpretation of gravity. The generalization allowed identifying Planck-scale constituent attributes from the universal constants of free space, like G and $\bar{h}$, that can be seen as unit conversion constants as a result. Second, as the effective field dynamics of the constituents must eventually obey Einstein’s field equations, a sub-quantum interaction law, although formulated within the emergent relativistic space-time framework, has been derived from an approximate solution of these equations.

Generalizing the workings of the holographic principle to all reference surfaces, however, also called for a corresponding generalization of the Bekenstein conjecture, which assesses the entropy change at a black hole’s surface upon mass aggregation. This conjecture has been used to connect the gravitational acceleration near a holographic surface to an entropy gradient by Padmanabhan [9] and Verlinde [10]. In this work, however, relating the experience of a distant mass to the entropy (gradient) has been achieved for non-holographic surfaces from the number of micro-constituents that are distributed over the surfaces’ fundamental degrees of freedom. Taking a Schwarzschild surface as reference immediately reproduced the holographic entropic gravity results and provided a constituent-based origin for thermodynamic black hole characteristics. The interpretation of gravity in terms of an effective constituent density gradient is believed to provide a more straightforward understanding towards an emergent quantum gravity theory.

The general conclusion “that acceleration is related to an entropy gradient” [9] or a constituent density gradient also calls for a more general interpretation of the fundamental forces. If reality is indeed identical to a single type of space-time micro-constituents interacting through the proposed law (or similar), than this assumption entails that not only effective space-time and gravity, but also the other fundamental forces should emerge from the interaction of the micro-constituents. Unruh’s argument that every acceleration induces a temperature was inverted by Padmanabhan [9] and Verlinde [10] to state that gravitational acceleration or inertia is induced by a temperature-induced entropy gradient, but can hence also be understood to be generally reversible, indicating that every fundamental acceleration (or force) is induced by an effective constituent density gradient.

In line with the common interpretation of Einstein’s field equations, one could indeed imagine that a composite body (i.e., a space-time constituent collection) experiencing no net force whatsoever must be located within an isotropic space-time constituent density distribution, while every “force” that disturbs the isotropy, as a “space-time curvature” effect on the surrounding micro-constituent density distribution, is compensated for by a macroscopic acceleration, as effectively induced by a sub-quantum micro-constituent dynamics according to Equation (30), to a geodesic trajectory. This view corresponds with the idea that according to general relativity gravity is not a force in the classical sense as objects do not couple to the gravitational field; objects just exist and, if not differently constrained, follow geodesic trajectories [36].

Differences between the Standard Model matter and force particles must in this view emerge from different types of ‘clustering’ of the space-time micro-constituents, while no specific clustering configuration seems to be required for the emergence of space-time and gravity. Note that correspondingly every part of the universe can be attributed mass and energy, but not any other Standard Model attribute that requires a specific constituent configuration. The strength gap between the gravitational pull and the other fundamental forces that involve clustered space-time anisotropies is therefore anticipated. In agreement with experiment, this gap however should narrow when the number of background constituents increases up to a high-energy level where the constituent density discrepancy becomes vague or disappears.

The biggest open question towards unification of the fundamental forces within this line of research is then whether the interaction according to the law proposed in Equation (30) also allows for different types of micro-constituent clustering behavior that yield Standard Model physics, or whether other constituent attributes and interaction laws are required. Yet, for the accustomed probability wave dynamics within quantum mechanics, one could expect that each constituent cluster shows an internal micro-constituent dynamics that can be assessed by the use of wave characteristics, which are merely descriptive choices in function of an observer’s Eigen-time. These descriptive choices could be quantized in terms of a wavelike Gibbs ensemble probability density function for the cluster’s micro-constituents. Thereby taking into account the finite extent $l_{P}$ of the constituents, one arrives at a canonical quantization that relates to quantum mechanics’ probability density function. This function is denoted “densité de présence” in French, wherefrom the (gravitational) “presence” attribute specification in this work.

## Figures and Tables

**Table 1:** Translation (first column) of universal constants of free space into space-time constituent attributes (second column) and its effect on the definition of basic units (third column).

Constants Translation	Constituent Attributes	Constituent Units
$\bar{h}=2G_{0}l_{P}^{2}\to l_{P}$	Size	$l_{0}=l_{P}$
c→c	Velocity	$t_{0}=t_{P}=l_{P}/c$
$G=c^{3}/2G_{0}\to G_{0}$	Gravitational presence	$m_{0}=G_{0}l_{P}/c=m_{P}/2$
$k_{B}=2S_{0}\to S_{0}$	Unit entropy	$S_{0}=S_{P}/2$ ($T_{0}=T_{P}$)
